# Lipopolysaccharide-Induced Cellular Activation May Participate in the Immunopathogenesis of Visceral Leishmaniasis Alone or in HIV Coinfection

**DOI:** 10.1155/2012/364534

**Published:** 2012-08-22

**Authors:** Joanna Reis Santos-Oliveira, Alda Maria Da-Cruz

**Affiliations:** ^1^Laboratório Interdisciplinar de Pesquisas Médicas, Instituto Oswaldo Cruz, FIOCRUZ, 21040-360 Rio de Janeiro, RJ, Brazil; ^2^Disciplina de Parasitologia, Faculdade de Ciências Médicas, UERJ, 21040-900 Rio de Janeiro, RJ, Brazil

## Abstract

Visceral Leishmaniasis (VL) is an infectious disease which constitutes a serious public health problem, integrating the list of neglected tropical diseases. The disease is characterized by a *Leishmania*-specific immune suppression T-cell depletion and a decrease of other hematopoietic cells. In parallel, an immunostimulatory response also occurs, represented by polyclonal B lymphocytes, T-cell activation, and systemic proinflammatory responses. Parasite antigens were believed to mediate both suppression and activation mechanisms, but these concepts are constantly being revised. Similar to reports on HIV/AIDS, we have proposed that gut parasitation by amastigotes and lymphocyte depletion could also affect gut-associated lymphoid tissue, leading to mucosal barrier breach and predisposing to microbial translocation. An increment of plasmatic lipopolysaccharide (LPS) levels observed in Brazilian VL patients was implicated in the reduced blood CD4^+^ and CD8^+^ T cell counts, systemic T-cell activation, pro-inflammatory cytokines and MIF plasma levels, suggesting that a bacterial molecule not associated with *Leishmania* infection can exert deleterious effects on immune system. Recent results also pointed that the proinflammatory response was potentiated in VL/HIV-AIDS coinfected patients. The LPS-mediated cell activation adds another concept to the immunopathogenesis of VL and can bring a rational for new therapeutic interventions that could ameliorate the management of these patients.

Visceral leishmaniasis (VL) is an infectious disease caused by protozoans of *Leishmania *sp. genus. VL is a serious public health problem integrating the list of neglected tropical diseases. In a recent World Health Organization report, 0.2 to 0.4 million cases were globally estimated in the last five years [1]. Ninety percent of them occur in only six countries: India, Bangladesh, Sudan, South Sudan, Brazil, and Ethiopia [1, 2]. The infection is transmitted by sandflies, and nowadays, *L.(Leishmania)donovani* and *L.infantum* (sin. *L.chagasi*) are the main species causing VL [3]. These protozoans are intracellular obligate parasites that infect macrophage cell lineages from lymphoid organs such as bone marrow, spleen, liver, and lymph nodes. VL is classically characterized by fever, hepatosplenomegaly, cachexia, blood cytopenia, and a high parasite burden [4, 5]. It has a high mortality rate, and even in treated patients, the case fatality rates are of 10–20% [1], especially in HIV-coinfected patients [6]. Immunological response is directly involved in the disease's clinical outcome, but the pathogenic mechanisms are still controversial, and the concepts are constantly being revised [7–11].

VL is classically considered an immune suppression-mediated disease characterized by T-cell depletion and decrease of other hematopoietic cells (erythrocytes, platelets, and neutrophils) [4]. Early studies from the 80s showed that active phase of VL evolves with an impairment of the specific effector T-cell response to leishmanial antigens, absence of a delayed-type hypersensitivity reaction to parasite antigens, and an inability of T lymphocytes to proliferate and produce interferon (IFN)-*γ* and interleukin (IL)-2 cytokine in response to leishmanial antigens [4, 12]. *In vitro* neutralization of IL-10 and IL-4 recovers the type 1 cytokine production in peripheral blood mononuclear cells (PBMC) stimulated with leishmanial antigens [13]. In accordance with this, restoration of the immune response to *L.infantum* is also achieved following specific treatment [13, 14] which reinforced the role of parasite antigens in the suppressive mechanisms [15]. However, other mechanisms have already been implicated in this immunodeficiency such as: circulating molecules acting as soluble receptors for IL-2 [16], immunocomplexes, lipoproteins [17], and deactivating cytokine interactions (IL-4, IL-10, and transforming growth factor [TGF]-*β*) [15]. These suppressive mechanisms are aggravated by the intense leucopenia especially on T-cell compartment, with a consequent decrease of both CD4^+^ and CD8^+^ T lymphocytes [9].

Although immunosuppression is considered a hallmark of acute VL, a remarkable polyclonal B-cell response also occurs. High titers of unspecific and specific immunoglobulins against leishmanial antigens were described [4], suggesting a dual role to parasite antigens, either lymphocytes' activation or inhibition. Therefore, others studies reported an intense release of inflammatory cytokines in the plasma of active VL patients, including tumour necrosis factor (TNF) [18], IL-6, IL-8, and macrophage migration inhibitory factor (MIF) [9, 10]. Besides, elevated levels of plasmatic IL-10 and TGF-*β* are also detected, showing that these patients present a mixed profile as a result of a cytokine storm [9, 19]. By consequence, VL clinically present as systemic inflammatory response syndrome [5] similar to sepsis, malaria, or dengue fever.

A systemic proinflammatory response is also observed in HIV-1/AIDS patients [20]. As the virus-mediated chronic immune stimulation does not fully explain the intense cellular activation, other mechanisms have been investigated. Of utmost impact, it was demonstrated that CD4^+^ T cell depletion from gut-associated lymphoid tissue (GALT) [21] leads to mucosal barrier breach and predisposing to microbial translocation (MT). Bacterial products as lipopolysaccharide (LPS) were responsible for an intense cellular activation and proinflammatory cytokine release due to its immunostimulatory activity [22].

Recently, Brenchley and Douek [23] reviewed all the restrictions that humans developed to protect themselves from intestinal microbiota translocation. In this context, any alteration in the gut selective permeability or loss of intestinal immune regulation can facilitate the MT. This phenomenon has been demonstrated in diseases involving the intestinal tract such as inflammatory bowel diseases [24] and graft-versus-host disease [25]. Taking into account that gut parasitation by *Leishmania* amastigotes is known to occur in VL [26, 27], it was also expected that a microbial leakage from gut into the circulation was likely to affect these patients. Moreover, VL shares similar pathogenic features to HIV/AIDS, most notably proinflammatory responses and systemic lymphocyte depletion [4]. Recently, our group demonstrated that the increment of plasmatic LPS levels observed in Brazilian VL patients was implicated in the reduced blood CD4^+^ and CD8^+^ T-cell counts, systemic T-cell activation, proinflammatory cytokine plasma levels, and higher MIF levels, suggesting that a bacterial molecule not associated with *Leishmania* infection can exert deleterious effects on immune system [9]. LPS levels are correlated to soluble CD14 (sCD14) and plasma intestinal fatty acid binding protein (IFABP) levels in VL patients. It implies that LPS was bioactive *in vivo*, probably having a luminal origin. An increase in activated status was shaped by increased percentages of activation-associated molecules (HLA-DR, CD38, and CD25) on T lymphocytes and high proinflammatory cytokines response. Consistent with this feature, LPS levels were positively correlated with IL-6, IL-8, and MIF. The hypothesis that bacterial products can impact on chronic immune hyperactivation status in VL prompted us to argue possible benefits of antimicrobial prophylaxis in conjunction with anti-*Leishmania* therapy. Thus, ongoing studies in experimental VL are being performed. 

Studies addressing MT in infectious diseases are scarce. To our knowledge, rather than HIV-1 infection [23] and VL [9], a LPS-induced immunostimulatory role was only demonstrated in HBV and HCV virus [28]. A spectrum of changes in the mucosal architecture can be observed in giardiasis, with consequent diarrhea episodes and adherence to the epithelium [29], raising the issue that any condition that causes gastrointestinal barrier damage may allow luminal contents leakage into circulation. In this context, intestinal parasitic disease such as amebiasis, strongyloidiasis, or criptosporidiasis, as well as gastroenteritis due to virus or bacteria could evolve with MT. These possibilities raise such pathogens, as candidates to further studies.

In the last 30 years, the expansion of the HIV/AIDS epidemic over leishmaniasis endemic areas and vice versa has increased the number of coinfected patients [6]. VL is an opportunistic disease in HIV/AIDS patients, although not yet considered an AIDS-defining disease. Both infections with *L.infantum* and HIV-1 share immune-compromising mechanisms that may affect the parasite control in VL coinfected patients [30]. Consequently, coinfected patients present a more severe disease in comparison to patients with VL alone, with increased parasite burden, drug resistance, and frequent relapses [6, 31]. On the other hand, *Leishmania* infection can also contribute to more rapid progression to AIDS, impairing both the lymphocyte depletion and the chronic immune activation, disturbances already observed in HIV-1-infected patients [32, 33].

Considering that MT is involved in activation mechanisms in HIV/AIDS patients and was also detected in VL, it was supposed that this phenomenon could be potentiated in coinfected patients. Recent studies (Santos-Oliveira et al., paper in preparation) showed that *Leishmania*/HIV-coinfected and HIV-1 monoinfected patients presented high LPS and IFABP levels, but the results were not statistically different. However, the plasma proinflammatory cytokines (IL-1*β*, IL-6, IL-8, IL-17, IFN-*γ*, and TNF) were much more higher in coinfected group. LPS levels along with immune consequences of *Leishmania* infection were associated with high levels of CD38 in T CD8^+^ in coinfected patients. These cofactors seem to contribute to the activation status by enhancing the plasma cytokine storm. The parasite influence in this system cannot be ruled out, although patients had experienced a clinical remission of VL symptoms after antileishmanial therapy.

In conclusion, the parasitation of intestinal mucosa and T-cell depletion can lead to GALT compromising, enabling microbial translocation of luminal gram-negative bacteria in VL. The LPS-mediated cell activation adds another concept to the immunopathogenesis of the complex viscerotropic *Leishmania*-host interaction. More importantly, immune activation can profoundly impact the VL clinical course and prognosis, contributing to increase the risk of death even under antileishmanial treatment. This mechanism is aggravated in *Leishmania*/HIV coinfected patients. These findings can bring a rational for new therapeutic interventions that could ameliorate the management of these patients, thus reducing the mortality of VL associated or not with HIV-1 infection.

## References

[1] Alvar J, Vélez I, Bern C (2012). Leishmaniasis worldwide and global estimates of this prevalence. *Plos One*.

[2] Maia-Elkhoury ANS, Carmo EH, Sousa-Gomes ML, Mota E (2007). Analysis of visceral leishmaniasis reports by the capture-recapture method. *Revista de Saúde Pública*.

[3] Kuhls K, Alam MZ, Cupolillo E (2011). Comparative microsatellite typing of new world *Leishmania infantum* reveals low heterogeneity among populations and its recent old world origin. *PLoS Neglected Tropical Diseases*.

[4] Saha S, Mondal S, Banerjee A, Ghose J, Bhowmick S, Ali N (2006). Immune responses in kala-azar. *Indian Journal of Medical Research*.

[5] Costa CHN, Werneck GL, Costa DL (2010). Is severe visceral leishmaniasis a systemic inflammatory response syndrome? A case control study. *Revista da Sociedade Brasileira de Medicina Tropical*.

[6] Alvar J, Aparicio P, Aseffa A (2008). The relationship between leishmaniasis and AIDS: the second 10 years. *Clinical Microbiology Reviews*.

[7] Carvalho EM, Teixeira RS, Johnson WD (1981). Cell-mediated immunity in American visceral leishmaniasis: reversible immunosuppression during acute infection. *Infection and Immunity*.

[8] Karp CL, El-Safi SH, Wynn TA (1993). In vivo cytokine profiles in patients with kala-azar. Marked elevation of both interleukin-10 and interferon-gamma. *Journal of Clinical Investigation*.

[9] Peruhype-Magalhães V, Martins-Filho OA, Prata A (2006). Mixed inflammatory/regulatory cytokine profile marked by simultaneous raise of interferon-*γ* and interleukin-10 and low frequency of tumour necrosis factor-*α*
^+^ monocytes are hallmarks of active human visceral Leishmaniasis due to *Leishmania chagasi* infection. *Clinical and Experimental Immunology*.

[10] Santos-Oliveira JR, Regis EG, Leal CRB, Cunha RV, Bozza PT, Da-Cruz AM (2011). Evidence that lipopolysaccharide may contribute to the cytokine storm and cellular activation in patients with visceral leishmaniasis. *PLoS Neglected Tropical Diseases*.

[11] Singh OP, Gidwani K, Kumar R (2012). Reassessment of immune correlates in human visceral leishmaniasis as defined by cytokine release in whole blood. *Clinical Vaccine Immunology*.

[12] Carvalho EM, Bacellar O, Barral A, Badaro R, Johnson WD (1989). Antigen-specific immunosuppression in visceral leishmaniasis is cell mediated. *Journal of Clinical Investigation*.

[13] Carvalho EM, Bacellar O, Brownell C, Regis T, Coffman RL, Reed SG (1994). Restoration of IFN-*γ* production and lymphocyte proliferation in visceral leishmaniasis. *Journal of Immunology*.

[14] Caldas A, Favali C, Aquino D (2005). Balance of IL-10 and interferon-*γ* plasma levels in human visceral leishmaniasis: implications in the pathogenesis. *BMC Infectious Diseases*.

[15] Goto H, Prianti MDG (2009). Immunoactivation and immunopathogeny during active visceral leishmaniasis. *Revista do Instituto de Medicina Tropical de São Paulo*.

[16] Barral-Netto M, Barral A, Santos SB (1991). Soluble IL-2 receptor as an agent of serum-mediated suppression in human visceral leishmaniasis. *Journal of Immunology*.

[17] Soares NM, Ferraz TPL, Nascimento EG, Carvalho EM, Pontes-de-Carvalho L (2006). The major circulating immunosuppressive activity in American visceral leishmaniasis patients is associated with a high-molecular weight fraction and is not mediated by IgG, IgG immune complexes or lipoproteins. *Microbial Pathogenesis*.

[18] Barral-Netto M, Badaro R, Barral A (1991). Tumor necrosis factor (cachectin) in human visceral leishmaniasis. *Journal of Infectious Diseases*.

[19] Nylén S, Maurya R, Eidsmo L, Das Manandhar K, Sundar S, Sacks D (2007). Splenic accumulation of IL-10 mRNA in T cells distinct from CD4^+^CD25^+^ (Foxp3) regulatory T cells in human visceral leishmaniasis. *Journal of Experimental Medicine*.

[20] Douek DC, Roederer M, Koup RA (2009). Emerging concepts in the immunopathogenesis of AIDS. *Annual Review of Medicine*.

[21] Brenchley JM, Schacker TW, Ruff LE (2004). CD4^+^ T cell depletion during all stages of HIV disease occurs predominantly in the gastrointestinal tract. *Journal of Experimental Medicine*.

[22] Brenchley JM, Price DA, Schacker TW (2006). Microbial translocation is a cause of systemic immune activation in chronic HIV infection. *Nature Medicine*.

[23] Brenchley JM, Douek DC (2012). Microbial translocation across the GI tract. *Annual Reviews of Immunology*.

[24] Harrison OJ, Maloy KJ (2011). Innate immune activation in intestinal homeostasis. *Journal of Innate Immunology*.

[25] Cooke KR, Olkiewicz K, Erickson N, Ferrara JLM (2002). The role of endotoxin and the innate immune response in the pathophysiology of acute graft versus host disease. *Journal of Endotoxin Research*.

[26] Baba CS, Makharia GK, Mathur P, Ray R, Gupta SD, Samantaray JC (2006). Chronic diarrhea and malabsorption caused by *Leishmania donovani*. *Indian Journal of Gastroenterology*.

[27] Luz KG, Tuon FF, Duarte MIS (2010). Cytokine expression in the duodenal mucosa of patients with visceral leishmaniasis. *Revista da Sociedade Brasileira de Medicina Tropical*.

[28] Sandler NG, Koh C, Roque A (2010). Host response to translocated microbial products predicts outcomes of patients with HBV or HCV infection. *Gastroenterology*.

[29] Müller N, Von Allmen N (2005). Recent insights into the mucosal reactions associated with *Giardia lamblia* infections. *International Journal for Parasitology*.

[30] Barreto-De-Souza V, Pacheco GJ, Silva AR (2006). Increased *Leishmania replication* in HIV-1-infected macrophages is mediated by tat protein through cyclooxygenase-2 expression and prostaglandin E 2 synthesis. *Journal of Infectious Diseases*.

[31] Cota GF, de Sousa MR, Rabello A (2011). Predictors of visceral leishmaniasis relapse in hiv-infected patients: a systematic review. *PLoS Neglected Tropical Diseases*.

[32] Santos-Oliveira JR, Giacoia-Gripp CBW, Alexandrino de Oliveira P (2010). High levels of T lymphocyte activation *in Leishmania-HIV-1 coinfected individuals* despite low HIV viral load. *BMC Infectious Diseases*.

[33] Alexandrino-de-Oliveira P, Santos-Oliveira JR, Dorval MEC (2010). HIV/AIDS-associated visceral leishmaniasis in patients from an endemic area in Central-west Brazil. *Memórias do Instituto Oswaldo Cruz*.

